# Immunologic Basis for Long HCDR3s in Broadly Neutralizing Antibodies Against HIV-1

**DOI:** 10.3389/fimmu.2014.00250

**Published:** 2014-06-02

**Authors:** Lei Yu, Yongjun Guan

**Affiliations:** ^1^Division of Basic Science and Vaccine Research, Institute of Human Virology, University of Maryland School of Medicine, Baltimore, MD, USA; ^2^Department of Microbiology and Immunology, University of Maryland School of Medicine, Baltimore, MD, USA

**Keywords:** VH replacement, HIV-1, broadly neutralizing antibodies, long HCDR3, immunologic mechanism, vaccines

## Abstract

A large number of potent broadly neutralizing antibodies (bnAbs) against HIV-1 have been reported in recent years, raising hope for the possibility of an effective vaccine based on epitopes recognized by these protective antibodies. However, many of these bnAbs contain the long heavy chain complementarity-determining region 3 (HCDR3), which is viewed as an obstacle to the development of an HIV-1 vaccine targeting the bnAb responses. This mini-review summarizes the current literature and discusses the different potential immunologic mechanisms for generating long HCDR3, including D–D fusion, VH replacement, long N region addition, and skewed D–J gene usage, among which potential VH replacement products appear to be significant contributors. VH replacement occurs through recombinase activated gene-mediated secondary recombination and contributes to the diversified naïve B cell repertoire. During VH replacement, a short stretch of nucleotides from previously rearranged VH genes remains within the newly formed HCDR3, thus elongating its length. Accumulating evidence suggests that long HCDR3s are present in significant numbers in the human mature naïve B cell repertoire and are primarily generated by recombination during B cell development. These new observations indicate that long HCDR3s, though low in frequency, are a normal feature of the human antibody naïve repertoire and they appear to be selected to target conserved epitopes located in deep, partially obscured regions of the HIV-1 envelope trimer. Therefore, the presence of long HCDR3 sequences should not necessarily be viewed as an obstacle to the development of an HIV-1 vaccine based upon bnAb responses.

## Introduction

The development of a protective HIV-1 vaccine is believed to be the best hope in the battle against HIV-1/AIDS. However, this goal remains elusive after 30 years of intense effort. Broadly neutralizing antibodies (bnAbs) against the HIV-1 envelope protein (Env) can be protective, as shown by passive immunization studies in non-human primates and humanized-mouse models (1–11). However, no HIV-1 vaccine candidate has been able to elicit a bnAb response. In the last 5 years, many novel bnAbs have been identified and are actively being pursued as templates for the rational design of an effective HIV-1 vaccine (12–20). Understanding the immunologic basis for the generation of these bnAb should help the design of an effective HIV-1 vaccine.

## HIV-1 Broadly Neutralizing Antibodies have Unique Features

Many potent bnAbs have been isolated and characterized from multiple subjects in the last 5 years (21–33), mainly due to the application of efficient methods for isolation of human monoclonal antibodies (mAbs) (27, 30, 34–37). These new HIV-1 bnAbs are much more potent and broader than previously described neutralizing Abs. With the elucidation of crystal structures of the HIV-1 Env trimer and gp120-antibody complexes (38–41), the vulnerable epitopes on the HIV-1 Env targeted by bnAbs are becoming clear. These new bnAbs can be categorized into four groups (Table 1). The first group is CD4 binding site (CD4bs) bnAbs represented by “VRC01-like” bnAbs (26, 28, 31, 32) that block Env binding to the primary receptor CD4. The second group includes the PGT series (29, 42–44), “PG9-like” bnAbs [PG9, PG16 (30), and CH01–04 (21)], which recognize both protein and glycan elements involving the V1V2 and V3 regions of gp120. The third group includes the recently described PGT151 series of bnAbs and the redefined 8ANC195 bnAb, which recognize glycan-related, gp120 and gp41 bridging regions (45–47). The fourth group targets the membrane-proximal external region (MPER) on gp41 and includes the antibodies 2F5, 4E10, 10E8, and M66.6 (24, 48, 49). These bnAbs collectively neutralize a majority of highly diverse HIV-1 strains. The new bnAbs and the recent crystal structure of HIV-1 Env trimer in complex with bnAbs have shed light on epitopes that could represent the basis for an Ab-based HIV-1 vaccine design. However, there are some common features of bnAbs that pose challenges to the development of a bnAb-based AIDS vaccine [reviewed in Ref. (20, 50) and Table 1].

**Table 1:** **Characteristics of the heavy chain V-gene of HIV-1 broadly neutralizing antibody**.

Category based on epitope cluster	HIV-1 bnAb		Heavy chain genes			HCDR3 (IMGT)		VH somatic mutation		Auto/poly reactivity**
	Class	Clone	V gene	D gene	J gene	Length	Potential VH replacement*	No. of nt (%)	No. of AA (%)	
CD4 binding site (CD4bs)	b12	b12	IGHV1-3	D1-1	J6*03	20	No	35 (12.2)	20 (20.8)	No
	VRC01	3BNC117	IGHV1-2	D6-25	J6*04	12	Yes	75 (26.0)	34 (35.4)	Yes
		3BNC60	IGHV1-2	D3-3	J6*04	12	Yes	82 (28.5)	38 (39.6)	Yes
		PG19	IGHV1-2	D6-25	J1*01	13	ND	66 (22.9)	30 (31.3)	NR
		VRC01	IGHV1-2	D2-21	J2*01	14	Yes	91 (31.6)	40 (41.7)	Neg.R
		VRC02	IGHV1-2	D5-12	J2*01	14	Yes	92 (31.9)	38 (39.6)	Neg.R
		VRC23	IGHV1-2	D5-24	J4*02	14	ND	62 (21.5)	30 (31.3)	NR
		PG20	IGHV1-2	D3-10	J1*01	15	ND	69 (24.0)	36 (37.5)	NR
		12A12	IGHV1-2	D4-17	J2*01	15	No	64 (22.2)	33 (34.4)	Yes
		12A21	IGHV1-2	D1-26	J2*01	15	Yes	60 (20.8)	30 (31.3)	Yes
		CH30	IGHV1-2	D3-16	J4*02	15	Yes	69 (24.0)	37 (38.5)	Neg.R
		CH31	IGHV1-2	D5-12	J4*02	15	Yes	72 (25.0)	37 (38.5)	Neg.R
		VRC03	IGHV1-2	D2-21	J4*02	16	Yes	85 (29.5)	39 (40.6)	Neg.R
		VRC-PG04	IGHV1-2	D2-8	J2*01	16	No	84 (29.2)	42 (43.8)	Neg.R
		VRC-PG04b	IGHV1-2	D2-15	J2*01	16	Yes	82 (28.5)	42 (43.8)	Neg.R
		VRC06	IGHV1-2	D2-21	J5*02	17	ND	88 (30.6)	46 (47.9)	NR
		NIH45-46	IGHV1-2	D1-26	J2*01	18	Yes	94 (32.6)	39 (40.6)	Yes
		3BC176	IGHV1-2	D5-12	J3*01	21	ND	69 (24.0)	34 (35.4)	Yes
		3BC315	IGHV1-2	D5-12	J3*01	21	ND	48 (16.7)	24 (25.0)	Yes
	8ANC131	8ANC131	IGHV1-46	D3-16	J6*01	18	No	74 (26.0)	38 (40.0)	Yes
		8ANC134	IGHV1-46	D3-16	J6*01	18	No	76 (26.7)	37 (38.9)	Yes
		1NC9	IGHV1-46	D5-24	J4*02	21	Yes	71 (24.7)	36 (37.5)	Yes
		1B2530	IGHV1-46	D3-10	J5*02	18	Yes	80 (27.8)	39 (40.6)	Yes
	CH103	CH103	IGHV4-61	D4-23	J4*01	15	ND	45 (15.8)	19 (20.0)	Yes
Glycan-dependent, V1/V2 and V3 related (QNE/supersite)	2G12	2G12	IGHV3-21	D1-26	J3*01	16	ND	61 (21.2)	31 (32.3)	Yes
	PGT145	PGT145	IGHV1-8	D4-17	J6*02	33	Yes	48 (16.7)	27 (28.1)	NR
		PGT141	IGHV1-8	D4-17	J6*02	34	Yes	46 (16.0)	27 (28.1)	NR
		PGT142	IGHV1-8	D4-17	J6*02	34	Yes	47 (16.3)	29 (30.2)	NR
	PG9	CH01	IGHV3-20	D3-10	J2*01	26	Yes	48 (16.7)	28 (29.2)	Neg.R
		CH02	IGHV3-20	D3-10	J2*01	26	ND	41 (14.2)	22 (22.9)	Neg.R
		PG9	IGHV3-33	D3-3	J6*03	30	No	40 (15.1)	18 (18.9)	Neg.R
		PG16	IGHV3-33	D3-3	J6*03	30	No	43 (14.9)	21 (21.9)	Neg.R
	PGT128	PGT135	IGHV4-39	D3/OR15-3a	J5*02	20	Yes	54 (18.6)	28 (28.9)	NR
		PGT137	IGHV4-39	D2-15	J5*02	20	Yes	67 (23.0)	32 (33.0)	NR
		PGT125	IGHV4-39	D3/OR15-3a	J5*02	21	Yes	60 (20.6)	28 (28.9)	NR
		PGT127	IGHV4-39	D3-16	J5*02	21	Yes	46 (15.8)	25 (25.8)	NR
		PGT128	IGHV4-39	D3-10	J5*02	21	Yes	59 (20.3)	29 (29.9)	NR
	PGT121	PGT121	IGHV4-59	D3-3	J6*03	26	Yes	56 (19.6)	23 (24.2)	NR
		PGT122	IGHV4-59	D3-3	J6*03	26	Yes	56 (19.6)	25 (26.3)	NR
		10-1074	IGHV4-59	D3-3	J6*03	26	ND	45 (15.8)	20 (21.1)	NR
	VRC24	VRC24	IGHV4-4	D3-9	J5*02	26	ND	64 (22.5)	29 (30.2)	NR
Glycan-related, gp120/gp41 bridging region	8ANC195	8ANC195	IGHV1-3	D3-3*01	J4*02	22	ND	80 (28.4)	40 (42.6)	Yes
	PGT151	PGT151	IGHV3-30*		J6*02	28	ND	60 (20.8)	27 (28.1)	Neg.R
		PGT152	IGHV3-30		J6*02	28	ND	56 (19.6)	29 (30.2)	Neg.R
		PGT154	IGHV3-30		J6*02	28	ND	53 (18.4)	25 (26.0)	NR
		PGT158	IGHV3-30		J6*02	28	ND	61 (21.2)	30 (31.2)	NR
gp41 MPER	MPER	4E10	IGHV1-69	D1-1	J4*02	20	ND	19 (6.6)	18 (18.8)	Yes
		2F5	IGHV2-5	D3-3	J6*02	24	ND	41 (14.1)	14 (14.4)	Yes
		10E8	IGHV3-15	D3-3	J1*01	22	ND	63 (21.4)	26 (26.5)	Neg.R
		M66.6	IGHV5-51	D3-10	J6*02	23	ND	11 (3.8)	9 (9.4)	Yes

*HIV-1 bnAb information was obtained from the Antibody Database, kindly provided by Dr. Anthony West (20) and the sequences were analyzed using IMGT V-QUEST (51)*.

*Potential VH replacement footprints were determined as reported (52). ND, not determined.

**Neg. R, negative in in vitro assay reported; NR, not reported. It should be noted that an in vivo test will be needed to determine a truly negative auto-/poly-reactivity as it was determined for b12. Although no direct auto-/poly-reactivity data for the PGT series bnAbs were reported, several representative antibodies of them (PGT121, PGT128, 10-1074, etc.) were shown to mediate effective protection in in vivo passive immunization studies, which indicates that they are likely negative in auto-/poly-reactivity.

The first is that the new HIV-1 bnAbs are highly somatically mutated, especially in the variable heavy chain region (VH) genes (21, 22, 26, 28–32, 53). This is in contrast with other human immunoglobulin G (IgG) antibodies and HIV-specific IgG antibodies with limited neutralizing activity (27, 54, 55). Many of the HIV-1 bnAbs also have insertions and deletions in their complementarity-determining regions (CDRs) (17, 26). This may reflect their prolonged, complex maturation path *in vivo* (17, 26, 56, 57), which would require extensive activity of activation-induced cytidine deaminase (AID) in germinal center B cells (58). Thus, induction of such highly somatically mutated antibody responses by vaccination is obviously a major challenge for bnAb-based HIV-1 vaccine development (20, 50).

The second feature is that many of the HIV-1 bnAbs are auto/poly reactive (26, 28, 31, 32, 59, 60). This might be a property acquired in the development of HIV-1 specific B cells during chronic HIV-1 infection that bypasses multiple B cell tolerance checkpoints (37, 61, 62). This phenomenon might be one of the reasons why a bnAb is usually generated after prolonged exposure to viral antigen in some HIV-1 infected people (26, 61, 62). Whether the auto/poly reactivity of these HIV-1 bnAbs is severe enough to prevent the induction of these antibodies *in vivo* in healthy individuals, which could be determined by *in vivo* testing of antibody gene knock-in animal models (63), will be critical to the success of a vaccine targeting these bnAbs (59). Alternatively, bnAbs with no or minimal auto/poly reactivity should be chosen as templates for HIV-1 vaccine (18, 24, 53, 61).

Another interesting feature is that many of the HIV-1 bnAbs have long (20–34 residues) heavy chain complementarity-determining region 3 (HCDR3) sequences (Table 1), especially in antibodies of the glycan-related V1/V2 and V3 category (Supersite group), the gp120/gp41 bridging region category and the gp41-MPER category. This contrasts with an average length of 16 residues of HCDR3 in human B cells (54). The HCDR3s of CD4bs bnAbs are relatively short (Table 1). The PG9-like and PGT128-like bnAbs in the Supersite group appear to have a long HCDR3 that can penetrate the glycan shield of the Env trimer and interact with the V1/V2 and/or V3 region of gp120. The new MPER targeting 10E8 also uses a long CDRH3 loop to reach the highly conserved hydrophobic residues on gp41 (42–44, 53). A bias against long HCDR3s during B cell development has been demonstrated in mice and rabbits (64, 65), which complicates using small animal species as an HIV-1 bnAb-based vaccination model (66). Although humans do generate antibodies with very long HCDR3s (67), the lower frequency of B cells encoding long HCDR3s and the potential bias of auto-reactivity were viewed as a challenge for eliciting bnAbs of long HCDR3s by vaccination due to the negative regulation of these antibodies during B cell development (14, 19, 37, 53, 64, 66). However, it should be noted that, although many long HCDR3 antibodies were reported to be auto-reactive and B cell precursors of auto-reactive antibodies are under negative selection during B cell development (37), the long HCDR3 and the auto-reactivity are two distinct aspects of antibodies. It is neither true that all long HCDR3 antibodies are auto-reactive, nor that all auto-reactive antibodies have long HCDR3s, though a long HCDR3 and auto-reactivity can sometimes be present in the same antibody. Data with HIV-1 bnAbs indicate that the negative selection against B cells encoding long HCDR3s is most likely a result of negative selection against auto-reactivity instead of the long HCDR3 itself. Many of the long HCDR3 bnAbs in the “Supersite” group of HIV-1 bnAbs and the PGT151 series bnAbs are not auto/poly reactive, while the CD4bs bnAbs group has many auto/poly reactive antibodies with shorter HCDR3s [Table 1 and review of (60)]. B cell precursors of non-auto-reactive long HCDR3 antibodies can pass negative selection checkpoints to become mature B cells. This view is strongly supported by the recent observation that long HCDR3s are present in significant numbers in the human naïve B cell repertoire and that they are primarily generated by the recombination events during B cell development (68).

Here, we review the current literature on the immunologic mechanisms for the generation of antibodies with long HCDR3s, among which potential VH replacement products appear to make a significant contribution in the generation of HIV-1 bnAbs. Our view is that, though negatively selected during B cell development, long HCDR3s are not necessarily an obstacle in the development of an HIV-1 vaccine targeting long HCDR3 bnAb responses.

## Immunologic Mechanisms for Generating Antibodies with Long HCDR3

HCDR3, a key determinant of antibody specificity (69), is a product of combinatorial rearrangement of the variable (V), diversity (D), and joining (J) gene segments. It is composed of the sequence from the V–D junction, the D region, the D–J junction and the 5′ end of the J gene. The alternative use of D reading frames, variation in junction sites due to P-nucleotides and addition of N-nucleotides, in addition to VDJ recombination and somatic hypermutation (SHM), contribute to HCDR3 diversity (70, 71). Secondary mechanisms of receptor editing/revision, gene conversion, and VH replacement also contribute to the HCDR3 diversity (72–75). Among the diversities of HCDR3, the length of HCDR3 can have a large impact on the function of the antibody repertoire and varies from mouse to human (64, 65). Four immunologic mechanisms have been described that can increase the length of HCDR3.

## Contribution of Somatic Hypermutation to Long HCDR3s

The accumulation of insertions introduced during the SHM process can theoretically increase the length of HCDR3 (76, 77). SHM related insertion/deletions (In/Dels) contribute substantially to the diversity of the human antibody repertoire, with an estimated frequency of 1.3–6.5% in circulating B cells, though short (1–2 residues) insertions are much more frequent than long insertions (77, 78). Interestingly, In/Dels from somatic mutation play a critical role in some bnAbs against HIV-1. The VRC01-like CH31-class bnAbs (Table 1) have a nine-residue insertion in H-CDR1 (32). The VRC06 bnAb has a seven-residue insertion in H-FR3 (33). The PGT128-class bnAbs have a 5–6 residue insertion in H-CDR2 (29). However, the contribution of SHM related insertion to long HCDR3s is hard to assign due to the complex nature of VDJ junctions. A convincing result from an in depth analysis of HCDR3 length by next-generation sequencing demonstrated that SHM typically does not alter the length of HCDR3 and long HCDR3s are not generated primarily through SHM related insertions (68).

## Long HCDR3s Usually Arise during VDJ Recombination

B cell precursors with long HCDR3s tend to be auto-reactive and are negatively selected during B cell development, which is a recognized mechanism for the bias against long HCDR3s in human mature B cell repertoire (37). However, deep sequencing the human HCDR3 repertoire revealed that long HCDR3s are present in the mature naïve B cell repertoire at a significant frequency (68). The naïve B cell pool contains 3.5% B cells of HCDR3s ≥24 residues and 0.43% B cells of very long HCDR3s (≥28 residues). The features of P- and N-addition length from VDJ recombination show positive correlations with increasing HCDR3 length. Further, the B cells encoding long HCDRs display biased germline gene usage. Long HCDR3s show a strong association with the use of the D2 (D2-2 and D2-15) and D3 (D3-3) gene families and the use of J6 gene segment. Interestingly, many of the HIV-1 bnAbs with long HCDR3s use these preferred D and J gene segments. The PG9-class and PGT121-class bnAbs use the D3-3 and J6 gene segments and show very long HCDR3s (Table 1). It should be noted that these long HCDR3-associated human D and J gene segments are substantially longer than other D and J gene segments (68). Small animals such as mice and rabbits do not have similar long D and J gene segments, which might be why they do not generate antibodies with long HCDR3s and why small animal species are not considered suitable as HIV-1 bnAb-based vaccination models (66). This further supports the idea that long HCDR3s are established in humans primarily during VDJ recombination before the antigen-driven affinity maturation process.

## D–D Fusion Recombinants Can Generate Long HCDR3s

D–D fusion is a V(DD)J recombination event that allows the generation of extremely long HCDR3s. D–D fusions are difficult to produce through normal V(D)J recombination because they violate the 12/23 rule (79). Although rare, these non-12/23 recombination events have been reported in *in vitro* and *in vivo* systems (80–82). High-throughput deep sequencing demonstrated that the frequency of D–D fusion in the naïve B cell population is about 1 in 800 naive B cells (79). The frequency is reduced in memory B cells. However, due to potential mismatches from somatic hypermutation, it is a challenge to accurately determine the frequency of D–D fusion in somatic-mutated memory B cells. The contribution of D–D fusion to long HCDR3s of HIV-1 bnAbs is unknown because almost all the bnAbs exhibit extensive hypermutation that make it hard to accurately match the germline D gene segments of HIV-1 bnAbs. HIV-1 bnAbs of PGT145 and PG9 classes (Table 1) have extremely long HCDR3s (34 and 30 residues, respectively) and are highly somatically mutated. IMGT junction analysis (51) of the HCDR3 of PGT145 reveals a 12 bp D4-17 sequence with three mismatches as well as an 11 bp D5-24 sequence with two mismatches, indicating that the long HCD3 of PGT145 might be the product of a D–D fusion. Therefore, it is possible that some HIV-1 bnAbs are derived from naïve B cells with D–D fusions.

## VH Replacement Contributes Significantly to Long HCDR3

VH replacement is a well-recognized mechanism of antibody gene rearrangement (73, 83). It occurs through recombinase activated gene (RAG)-mediated secondary recombination (84) and contributes to the diversified naïve B cell repertoire (85). It is a process in which secondary V–V(D)J recombination results in replacement of the variable gene while preserving the original D–J recombination. It appears to occur early in B cell development as a mechanism to rescue non-functional and unwanted IgH genes to further diversify the IgH repertoire (86–88). The secondary recombination during VH replacement involves a cryptic recombination signal sequence (RSS) within a previously rearranged V(D)J joint with a 23 bp RSS from an upstream invading VH gene (86). During this process, a short stretch of nucleotides from previously rearranged VH genes are left within the newly formed HCDR3 and, therefore, elongate the HCDR3 region and provide a potentially identifiable “footprint” of VH replacement (75, 89).

By footprint analysis, the frequency of VH replacement in normal peripheral B cells was estimated to be 5.7% (52), which is significantly higher than that of D–D fusions. Although not all VH replacements necessarily result in VH genes with long HCDR3s, a high frequency of anti-HIV antibodies contain potential VH replacement footprints and many of these antibodies also have long HCDR3s (52). Seventy-three percent of anti-HIV CD4 induced (CD4i) antibodies and all PGT-class bnAbs (Table 1) contain VH replacement footprints. Both CD4i and PGT antibodies tend to be encoded by IgH genes of long HCDR3s, which are used to reach recessed regions of the Env (39, 52). These observations indicate that VH replacement may contribute significantly to HIV-1 antibodies that use long HCDR3s.

However, the detection of VH replacement by footprint analysis is controversial. Footprint determination of VH replacement could result in false positives because footprints can be mimicked by processes other than VH replacement, such as N-addition (72, 90). It could also result in false negative sequences because not every VH replacement products will have a detectable footprint (85, 90). Yet, footprint analysis is currently the only available choice for VH replacement studies on human primary samples and there is no question that VH replacement can generate antibodies with long HCDR3s.

## Discussion

Three of the four potential immunologic mechanisms for the generation of antibodies with long HCDR3s occur mainly at the time of V(D)J recombination during primary B cell development. There are 3.5% B cells with HCDR3s ≥24 amino acid residues and 0.43% B cells with very long HCDR3s (≥28 residues) in the naïve B cell population (68). This is a significant number when one considers the total of more than 10^12^ potentially different antibodies in the human B cell repertoire. Therefore, long HCDR3s, while relatively low in frequency, are a normal part of the naïve B cell repertoire that can actively participate in humoral immune responses. B cells with long HCDR3s appear to be selected by Env antigens to generate HIV-1 bnAbs targeting conserved epitopes located within deep regions of the HIV-1 envelope trimer. Long HCDR3s alone should not necessarily be viewed as an obstacle to the development of an HIV-1 vaccine targeting the long HCDR3 bnAb responses. Yet, how to induce highly mutated and auto-reactive HIV-1 bnAb response remains a true challenge for HIV-1 vaccine development (60).

The high frequency of VH replacement footprints in many HIV-1 bnAbs suggests a new strategy for HIV-1 vaccine development; we should first understand the mechanism regulating VH replacement events during B cell development (90, 91) and then find a safe procedure to increase the frequency of VH replacement events before immunization. This strategy should increase the frequency of long HCDR3 germline B cells of HIV-1 bnAbs in the naïve B cell pool, which, in turn, may improve the potential of generating bnAb responses against HIV-1. Increasing the frequency of long HCDR3-containing B cells through manipulating the level of VH replacement may lead to more opportunities in generating bnAbs of long CDRH3s. But this remains to be tested because increasing the frequency of HIV-1 bnAbs’ germline B cells may not be sufficient to generate bnAb responses.

Recent studies on the generation of HIV-1 bnAbs in HIV-1 infected individuals have highlighted the co-evolution of the HIV-1 Env diversity and the breadth of neutralizing antibody responses against Env (26, 56, 57, 92), which indicates an antigen-driven pathway for HIV-1 bnAbs. Since it was demonstrated that Envs from different HIV-1 strains are not equal in activating HIV-1 bnAbs’ germline B cells (26, 57, 93), a proper Env antigen with the right conformational epitopes may be required to activate HIV-1 bnAb germline B cells (61, 94) that presumably exist in most healthy individuals. Many of the HIV-1 bnAbs with long HCDR3s, such as PG9 and PGT151, recognize conformational epitopes that are not well exposed in recombinant gp120 or gp140 (30, 46). Therefore, the construction of recombinant Env proteins of native gp140 trimers (39) and/or constrained gp120s (95) that can preferentially expose epitopes recognized by bnAbs would be good antigen candidates in this regard. Further, a proper immunization strategy, such as sequential immunizations with selected diverse Env antigens and proper follicular helper T cells, will likely be required to drive the antibody responses toward highly mutated bnAbs (17, 20, 50).

## Conflict of Interest Statement

The authors declare that the research was conducted in the absence of any commercial or financial relationships that could be construed as a potential conflict of interest.
